# Effect of Baduanjin exercise on primary osteoporosis: study protocol for randomized controlled trial

**DOI:** 10.1186/s12906-023-04161-y

**Published:** 2023-09-16

**Authors:** Chuanrui Sun, Ming Chen, Xiaoyang Wang, Baoyu Qi, He Yin, Yingxia Ji, Na Yuan, Shangquan Wang, Liguo Zhu, Xu Wei

**Affiliations:** 1 Wangjing Hospital, China Academy of Chinese Medical Sciences, Beijing, China; 2 The First Clinical Medical College, Shaanxi University of Traditional Chinese Medicine, Xi’an, China

**Keywords:** Osteoporosis, Baduanjin exercise, Osteoporotic pain, Balance ability

## Abstract

**Background:**

Lack of exercise is often a major cause of chronic disease. Osteoporosis (OP) is a chronic disease with multifactorial co-morbidity. Baduanjin (BDJ) exercise may be a powerful tool for modifying risk factors. The aim is to provide more evidence about the effectiveness of BDJ exercise in improving pain and balance ability in patients with OP.

**Methods:**

In the prospective randomized controlled trial, 160 participants will be recruited and randomized to the treatment group (BDJ exercise combined with Calcium carbonate and D3) or the control group (Calcium carbonate and D3) at 1:1 ratio. Participants in the treatment group will receive 24-week BDJ exercise for 30–60 min, 3 times a week, along with Calcium carbonate and D3 at each day, while participants in the control group will receive Calcium carbonate and D3 only. All outcome indicators will be measured at baseline, after the 6th month of treatment and 6th month after the end of treatment. The primary outcomes include pain and balance ability, as measured by the visual analogue scale (VAS) and Berg balance scale (BBS). The secondary outcomes will primarily include bone mineral density (BMD), laboratory tests (including P1NP, β-CTX, MSTN, FDF-23, NPY), the timed “up and go” (TUG) test, the morse fall scale (MFS), the five-times sit-to-stand test (FTSST).

**Discussion:**

The study will hopefully confirm that BDJ exercise, as a non-drug intervention, should be recommended for patients with OP to prevent bone loss, falls and fractures.

**Trial registration:**

International standard randomized controlled trial number (ISRCTN) registry: ISRCTN76945140 registered on 07/06/2022.

## Background

Osteoporosis (OP) is a physiologically degenerative disease that occurs with age, often accompanied by a decrease in bone strength and an increased risk of low-energy or fragility fractures [1, 2]. The global prevalence of OP in the elderly was 21.7%, and the prevalence rates in Asia, America, and Europe were 24.3%, 11.5%, and 16.7%, respectively, with the highest in Asia [3]. According to a recent epidemiological survey about OP in China, the prevalence rate of men over 40 years old was 5%, and that of women over 40 years old was 20.6% [4]. Obviously, the situation is heavily serious, which poses a great challenge. OP, as a multifactorial co-causative chronic disease, has diverse approaches to prevention and treatment.

Lack of exercise is often the main cause of chronic diseases [5], and various forms of exercise have also become a sharp edge in fighting against diseases, which can be well balanced in both disease prevention and treatment [6]. At the same time, exercise is considered the most effective management method to improve physical function and mental health in the elderly, and widely recommended to improve bone health [7, 8]. In addition, various guidelines also suggest that a multi-component exercise program should be explored with OP, including resistance training, balance exercises, and aerobic physical activity [9–11]. Traditional Chinese exercises have been inherited and reformed, which combines the concepts of multicomponent movements, and play a significant role in combating the occurrence and development of diseases.

Baduanjin (BDJ), defined as an energy-burning exercise with traditional Chinese medicine characteristics, may be a powerful tool for adjusting risk factors about OP. This is a safe combination of aerobic and resistance exercise, consisting of eight movements that are less physically and cognitively demanding, easier to learn and practice, less restrictive and safe [12]. Therefore, BDJ exercise is a very suitable choice for the elderly because it can enhance body function by coordinating posture, movements, breathing and meditation throughout the body. Previous series studies have shown that BDJ exercise can provide physiological benefits, including improving cardiovascular risk factors [13, 14], quality of life [15], and mental health [16]. In China, BDJ exercise has become a popular, safe, and health-promoting form among the elderly.

Bone mineral density (BMD) is the gold standard for OP, however, one of the characteristics of OP is pain and the serious outcome is fracture, and then, balance ability is closely related to the occurrence of fracture [9]. Therefore, the study focused on pain and balance ability, two key factors associated with the development of OP. At first, we conducted a systematic review and meta-analysis in order to initially explore the therapeutic effect of BDJ exercise on OP [17], and the results suggested that it might be beneficial to improve BMD, relieve pain, improve balance ability, influence serum bone gla protein (BGP) and alkaline phosphatase (ALP) in patients with OP, and we also found previous studies to be of low quality. Therefore, we will plan to conduct a prospective superior randomized controlled trial and further determine the effectiveness of BDJ exercise on pain and balance improvement, which can provide a broad perspective in relationship between BDJ and OP.

## Methods

### Study aim and design

**Fig. 1:**
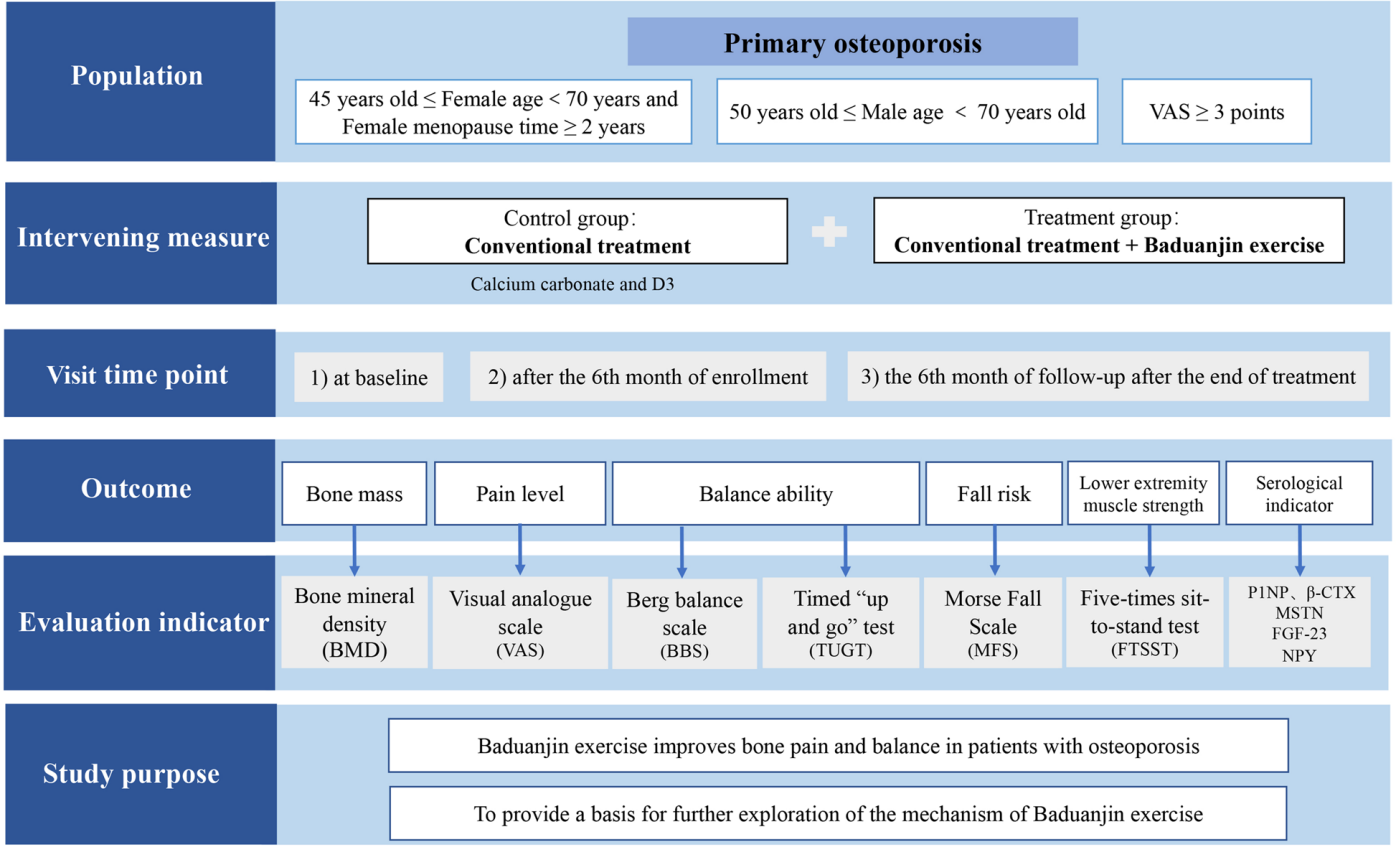
Flow diagram of the study

**Fig. 2:**
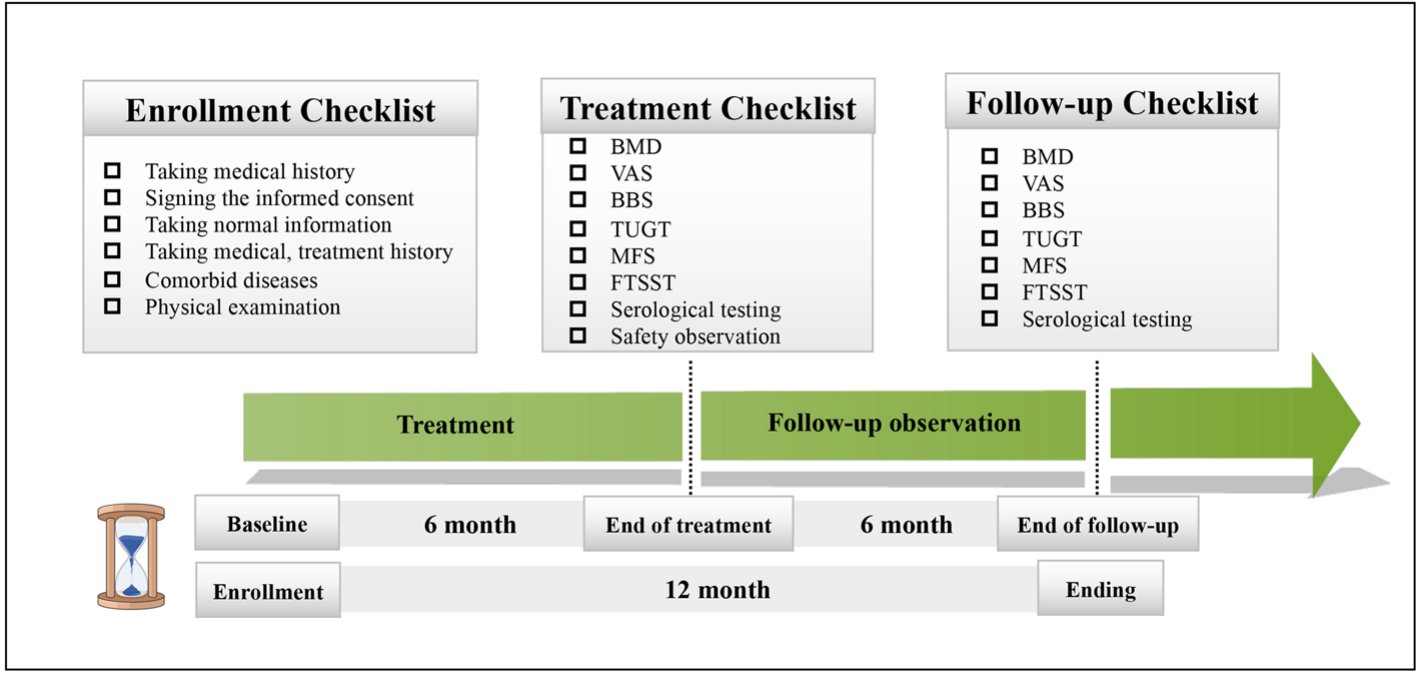
Outcome evaluation schedule

The protocol design is a two-arm parallel randomized controlled trial with allocation concealment and blinding of the assessors. Participants or the public were not involved in the design, or conduct, or reporting, or dissemination plans of our research. The procedure and outcome evaluation schedule for this trial are shown in Figs. 1 and 2.

### Study population

The participants are OP patients, with no gender restriction. The diagnostic, inclusion, exclusion, and withdrawal criteria are as follows.

1. Diagnostic criteria
  Referring to the *Chinese Guidelines for the Diagnosis and Treatment of Primary Osteoporosis* (2022) [18] and WHO [19], the diagnosis is mainly based on the BMD of dual energy X-ray absorptiometry (DXA). Based on DXA results, the study will select T-value ≤ -2.5 as the subject screening criterion. DXA will be operated by trained imaging technicians assigned by each sub-center.
2. Inclusion criteria
  Eligible participants meet the following criteria: 1) Primary OP: T-value ≤ -2.5. 2) Female aged from 45 to 70 years old and menopause time is greater than 2 years, which are met at the same time. Male aged from 50 to 70 years old. 3) Visual analogue scale (VAS) is greater than or equal to 3 points. 4) Patients who are willing to participate in the study.
3. Exclusion criteria
  The exclusion criteria are as follows: 1) Lumbar fusion or severe degeneration hinders BMD measurement, and there are less than two continuous intact lumbar vertebrae that can be evaluated by DXA. 2) Patients with serious primary diseases, such as cerebrovascular, digestive tract, and lung diseases, because these clinical manifestations may interfere with the results of the study. 3) Secondary OP: abnormal bone mass caused by similar diseases, including diabetes, thyroid disease, Cushing's syndrome, rheumatoid arthritis, multiple myeloma, gout, malabsorption syndrome, systemic lupus erythematosus, etc. 4) The participants have taken drugs that affect bone metabolism within 3 months before enrollment, such as bisphosphonates, glucocorticoids, calcitonin, anticonvulsant drugs, heparin, etc. 5) Those with a history of severe mental illness or poor compliance. 6) Allergic constitution. 7) Those who have participated in other clinical trials within 3 months.
4. Withdrawal criteria and management
  Participants will be allowed or required to withdraw for the following reasons: 1) Violation of the protocol. 2) Severe illness that prevents continuation. 3) Serious adverse events during the study period. 4) Voluntarily asked to withdraw from the trial. 5) Lost to follow-up. 6) Using drugs that may affect bone metabolism during the period.

### Recruitment

Participants will be recruited from hospitals or communities in Beijing, Tianjin, and Shaanxi province, China. The recruitment form is mainly a combination of online and offline methods, including Internet publicity, and setting up on-site recruitment stations, etc. First, potentially eligible individuals will complete doctors screening to determine their eligibility based on inclusion and exclusion criteria. Qualified individuals interested in participating in the study will have an informed discussion with trained research assistants. Research assistants will obtain Informed Consent before starting the baseline assessment.

### Randomization

Participants who meet the inclusion and exclusion criteria will be randomly divided into the control group and the treatment group, with 80 patients in each group. Due to the peculiarities of BDJ exercise itself, we could not blind participants or trainers, but outcome assessors and statisticians will be blind. The research will entrust a professional data management department to generate random numbers system (http://112.126.58.121:8082/bdj) and monitor trial quality. According to the group designated by the random number, research assistants accurately record the participants' random number, group, and intervention.

### Intervention

During the trial period, participants in the treatment group will be given BDJ exercise combined with Calcium carbonate and D3, and in the control group will be only given Calcium carbonate and D3. The intervention period will last 24 weeks. The requirements for the BDJ exercise will be as follows.

1. Preparatory stage (the first 2 weeks, mastering movements stage)
  Participants need to be organized to practice BDJ exercise for 3 times a week. The researchers will hire professional coaches to guide and correct the movements. During rest time, research assistants will distribute free videos to practice by themselves at home.
2. Conventional stage (the last 22 weeks, regular exercise stage)
  The participants need to exercise 3 times a week, 30 to 60 min each time, and the amount of exercise may be appropriate as long as the participants do not experience fatigue. The research assistants will lead exercise once a month, and the rest time will be practiced by themselves. The researchers will give monthly follow-up calls or home visits to record the completion and adjust the exercise frequency according to the actual conditions. During the trial period, the researchers will instruct the participants to avoid other physical activities as much as possible, for example, swimming. If there are associated exercises, the research assistant needs to record details.
  We will recommend the participants form a small team to exercise together with their familiar people, and distribute free brochures, BDJ exercise teaching videos and special lectures about OP at different periods, so as to maximize enthusiasm and compliance, which can ensure the study completion to the greatest extent.

### Outcome assessment

Variables in this study included basic information, clinical characteristics, adverse events, primary and secondary outcomes. We will collect basic information and clinical characteristics at baseline (1 week before randomization). Primary and secondary outcomes will be measured at baseline and after the 6th month of treatment (6 months after enrollment). In addition, follow-up assessments will be conducted at 6th month after the end of treatment (12 months after enrollment). All primary and secondary outcomes will be assessed by professionally qualified doctors who will be unaware of assigned outcomes.

1. Basic information and clinical characteristics
  Research assistants will use the Case Report Form (CRF) designed for this study to collect basic information and clinical characteristics, including gender, age, education, height, weight, waist circumference, hip circumference, co-morbid history, personal life history and medication use. Body mass index (BMI) was calculated from height and body mass, and waist-to-hip ratio was calculated from waist circumference and hip circumference.
2. Primary outcomes
  Pain induced OP is measured using the visual analogue scale (VAS, 0–10 points). 0 point represents no pain, 1–3 points are mild pain, 4–6 points represent moderate pain, although the pain is strong but still tolerable, 7–10 points represent severe pain, which gradually reaches difficult degree of tolerance.
  Balance ability is measured using the Berg balance scale (BBS, 0–56 points). The higher the score, the better the balance ability. At the same time, the risk of falling can be assessed according to the results: BBS < 40 points means at risk of falling.
3. Secondary outcomes
  Bone mineral density (BMD) is measured using DXA. Fixed professionals are responsible for the verification and measurement of the machine, and the inspection parts mainly include lumbar vertebrae (L1-L4) and bilateral hips.
  Laboratory tests (blood serum) are measured with Luminex Assay. With reference to the Recommended Experiments for Biochemical Indicators of Bone Metabolism (2020 Expert Consensus, China) [20], the preliminary research results, and the literature survey, The serological indicators include Type I Procollagen N-terminal Propeptide (P1NP), β-collagen Degradation Product (β-CTX), Myostatin (MSTN), Fibroblast Growth Factor-23 (FGF-23), and Neuropeptide Y (NPY). Qualified researchers will draw 5 ml of venous blood from the participants in the morning on an empty stomach, and complete the aliquots within 30 min.
  Fall risk is measured with the timed “up and go” (TUG) test and morse fall scale (MFS). Research assistants need to record the time, the participant's gait, and the falling risk according to the following criteria: 1 point is normal, 2 points are very slightly abnormal, 3 points are mildly abnormal, 4 points are moderate abnormality, 5 points are severe abnormality. The MFS was developed by Professor Janice Morse in 1989. The content of the scale evaluation involves the history of falls in the past 3 months, gait, cognition, etc. The total score is 125 points, and the higher the score, the greater the risk of falling. A score greater than 45 is considered high risk of falling, 25 to 45 is considered medium risk, and less than 25 is considered low risk.
  Lower extremity muscle strength is measured with five-times sit-to-stand test (FTSST). The assistants need to record the total time spent. At last, the test was performed 3 times and the final average was taken.

### Safety measurements

Research assistants will monitor participants for adverse events during the treatment. Research assistants guide the preparation and relaxation exercises before and after the practice to prevent the elderly from being injured. During the trial period, the BDJ exercise should be stopped if serious complications, other diseases, or adverse reactions occur. Meanwhile, corresponding treatment measures should be taken. Any adverse events that occur will be reported immediately and in detail to the research center that will assess the causal relationship and severity associated with the intervention. Serious adverse events will be reported to the ethics committee to decide the next steps.

### Sample size

According to the existing results combined with expert experience [21, 22], we assume α = 0.05 and β = 0.10 based on an allocation ratio of 1:1, then the sample size is 64 cases respectively in the two groups, totaling 128 cases according to VAS. Considering the 20% dropout rate, a total sample size of 160 cases is required. Then, we also calculated the sample size to be 146 cases according to BBS. Finally, we set the sample size of this study to 160 cases.

### Data collection and management

Data will be recorded on CRF, which will be reviewed and validated by statistical experts for final analysis. First, it is converted into electronic data through the EpiData (EpiData Association, Denmark) by two independent research assistants after the consistency of data is checked in CRF. When all information is finally confirmed, the electronic database will eventually be locked.

### Statistical analysis

All statistical analyses were performed using SPSS 25.0 and Python 3.11. Statistical significance was set at a P value less than 0.05. Continuous variables that conform to a normal distribution are represented by the mean and standard deviation, and variables that do not conform to a normal distribution are represented by the median and interquartile range. Categorical variables will be described as frequencies or percentages. Missing data will be filled in using multiple imputation.

To account for correlation among repeated measurements, intervention effects on the primary and secondary outcomes were examined by generalized estimating equations (GEE) with baseline measurement as the covariates. The group-by-time interaction, which indicated the difference for a given outcome between interventions over time, was considered our primary measure of intervention effects.

Adverse effects will be analyzed using the χ^2^ test or Fisher's test. If it cannot run a normal analysis, we will choose descriptive analysis for presentation.

## Discussion

As an integrated mind–body exercise, BDJ is less physically and cognitively demanding than Tai Chi. A recent meta-analyses suggested that mind–body exercise might be the best exercise type to increase BMD in the lumbar spine and femoral neck [23]. Exercise causes gravitational and muscle-contractional loads on the bones, and lack of exercise is often the main cause of bone loss in weight-bearing bones [5]. Therefore, the stress stimulation of BDJ exercise on the bones determines that it could be an effective treatment for OP.

The biggest challenge is compliance in this study. Even more frightening are concerns about adherence to OP treatment, compared with the consequences of low bone mass. It is estimated that more than 70% of individuals at risk of OP who are on treatment do not receive continue treatment beyond the first year [24]. Active exercise is far more effective than passive therapy. Exercise training is more effective than actual therapist therapy in reducing pain, improving physical function, and mental health [25]. It is far from enough to passively promote their awareness only by external intervention, and every elder person needs to pay more attention. Patient’s adherence about exercise programs is known to be a difficult area in clinical practice. Many patients do not follow clinician recommendations and are at risk of worsening their condition [3]. We are also actively preparing for this issue, for example, we encourage community residents who are familiar with each other, which is also convenient for us to manage. At the same time, we will establish an online communication group and organize community lectures to deal with such situations.

However, there are several limitations in our study. Firstly, there are no guidelines to reconsider the most appropriate type, frequency, intensity and duration of physical activity in patients with OP to prevent bone loss and fracture from a clinical perspective [6]. Therefore, we can only explore the optimal frequency and intensity of BDJ exercise based on current experience and experts’ recommendation. Secondly, how to coordinate breathing, posture and thinking is the key when practicing BDJ exercise. It requires the mind, body and breathing to participate in the training at the same time, in order to achieve the coordination and unity of the whole body, as well as the desired results to the greatest extent. Moreover, the effect may not be ideal, if it is not practiced correctly and if the practitioner does not have a proper grasp of the inherent nature. Therefore, we try to avoid it as much as possible by resorting to more guidance and explanation from professionals, but it is undeniable that it does exist.

The study will investigate the effectiveness of BDJ exercise on improving pain and balance performance in patients with OP. The results hopefully confirm that BDJ exercise, as a non-drug intervention, should be recommended for patients with OP to prevent bone loss, falls and fractures. The ultimate goal is to translate our findings into concrete recommendations that can be used in clinical practice. In addition, the findings may provide a basis for further exploration of the mechanism of BDJ exercise.

## Data Availability

The data sharing plans for the current study are unknown and will be made available at a later date.

## Abbreviations

OP: Osteoporosis
BDJ: Baduanjin
RCT: Randomised control trial
VAS: Visual analogue scale
BMD: Bone mineral density
BBS: Berg balance scale
TUG: The timed “up and go” test
MFS: Morse fall scale
FTSST: Five-times sit-to-stand test
DXA: Dual energy X-ray absorptiometry
CRF: Case report form
BMI: Body mass index
P1NP: Type I procollagen N-terminal propeptide
β-CTX: β-Collagen degradation product
MSTN: Myostatin
FGF-23: Fibroblast growth factor-23
NPY: Neuropeptide Y
BGP: Bone gla protein
ALP: Alkaline phosphatase
